# Strengthening the meaning in life among college students: the role of self-acceptance and social support - evidence from a network analysis

**DOI:** 10.3389/fpsyg.2024.1433609

**Published:** 2024-07-15

**Authors:** Caiyun Wu, Xueting Liu, Jinxin Liu, Yanqiang Tao, Yuting Li

**Affiliations:** ^1^Center for Psychological Education and Counseling, Southwest University of Science and Technology, Mianyang, China; ^2^Department of Student Affairs Management, Mudanjiang Normal University, Mudanjiang, China; ^3^School of Computer Science and Technology, Southwest University of Science and Technology, Mianyang, China; ^4^Faculty of Psychology, Beijing Normal University, Beijing, China; ^5^Department of Chinese Medicine Nursing, School of Nursing, Anhui University of Chinese Medicine, Hefei, Anhui, China

**Keywords:** self-acceptance, social support, meaning in life, college students, network analysis

## Abstract

Meaning in life, which has two possible sources: self-acceptance and social support, is essential to the mental health and development of college students. The current study aims to further clarify the symptom-level relations between meaning in life, self-acceptance, and social support, finding possible ways to improve meaning in the life of college students. Thousand three hundred and forty-eight Chinese college students completed the online questionnaire, including Self-acceptance Questionnaire, Social Support Rating Scale, and Meaning in Life Questionnaire and the data from 1,263 participants was used. Cross-sectional network analysis was used to examine the relation between self-acceptance and social support. We also explored the relation between dimensions of self-acceptance and social support and meaning in life using the flow network. The results show symptom “SlA” (self-acceptance) is the bridge symptom linking self-acceptance and social support. In the flow diagrams, “SlA” is directly and positively associated with the presence of meaning. Objective Support shares the strongest positive association with the search for meaning. The symptom “SIA” may be an important targeting symptom when trying to improve the meaning in life of college students. Additionally, social support is essential for college students to develop meaning in life.

## 1 Introduction

Meaning in life is an important yet declined mental resource for the well-being and comprehensive development of college students (Huang et al., 2022; Olstad et al., 2023). Self-acceptance and social support may serve as two possible sources from which college students may attain more meaning in life (King and Hicks, 2021). To prompt the exhibition of meaning in life among college students, the relation between meaning in life, self-acceptance, and social support needs to be clarified.

### 1.1 Meaning in life for college students

Meaning in life, characterized as the concerns with the core significance and purpose of the personal existence of an individual, contains two factors, the presence of meaning and the search for meaning (Steger et al., 2006). The presence of meaning refers to the subjective sense that one’s life is meaningful whereas the search for meaning implies the drive and orientation toward finding meaning in one’s life, both of which are significant to one’s well-being and personal achievement (Dezutter et al., 2013). As Frankl (1992) argued, human beings are characterized by a “will to meaning,” characterized as the forceful drive to search for meaning and significance in their life, and failing to achieve meaning can lead to psychological distress. Additionally, Maslow (1971) also stated in his theory of a hierarchy of needs that meaning in life is important for individuals to maximize their full potential and attain self-fulfillment. In line with the theory, a meta-analysis conducted on 45 published papers also found that people with a higher level of meaning in life tend to experience more subjective well-being (Jin et al., 2016). And multiple longitudinal analyses also showed that greater meaning in life could precisely predict more life satisfaction, decreased and less severe internalizing problems (such as depression) and externalizing problems (such as problematic internet use), as well as higher quality of life (Liu M. H. et al., 2021; Dewitte et al., 2022; Fischer et al., 2023; Wang et al., 2023).

Noticeably, meaning in life can be extraordinarily critical to college students. College years have long been seen as an important period for the adaptation and transformation into an independent and capable individual (Medalie, 1981). As Fisher and Noble (1960) indicated, in college, students are confronted with the critical developmental tasks of achieving identity, which consist of achieving emotional independence, selecting and preparing for occupation, and exhibiting a scale of values and ethics system to live by. During this period, meaning in life can serve as an important mental resource that keeps students continuously reaching for the achievement of identity (Olstad et al., 2023). Indeed, the loss of meaning in life may result in failures in completing developmental tasks, which could manifest as adaptation problems, increased mental problems including depression, anxiety and social media addiction, as well as worsened academic performance, greatly hindering the self-fulfillment and overall well-being of college students (Cho, 2021; Ye et al., 2021; Baquero-Tomás et al., 2023; Guo Y. G. et al., 2023; Zhao, 2023). Alarmingly, it has raised much attention that the loss of meaning in life, or the so-called “hollow-heart disease” is becoming increasingly prevalent among Chinese college students (Huang et al., 2022). Therefore, it is of great necessity to identify effective ways to assist college students exhibit and maintain meaning in life.

Previous research has documented the significant positive impacts of meaning in life, no matter among the general or college students. However, it should be noted that even though presence of meaning and search for meaning are two components of meaning in life, the relation between presence of meaning and search for meaning is not a simply positive association. Two models were proposed and they were the Presence-to-Search Model (people with low levels of presence of meaning will search for meaning) and the Search-to-Presence Model (people who search for meaning will experience greater meaning) (Steger et al., 2008). Lots of studies have supported the Presence-to-Search Model (Steger et al., 2008). In terms of Search-to-Presence Model, even though plenty of studies have identified that search for meaning may not lead to higher presence of meaning (or having significant but weak relationship) (Dezutter et al., 2015; Newman et al., 2018), seems opposing to the perception of Search-to-Presence Model, there are also research suggested that searching for meaning was positively related to presence of meaning among individuals with greater maladaptive traits (Chu and Fung, 2021). Thus, more studies are needed to explore the discrepancies and complex relation between search for meaning and presence of meaning.

### 1.2 Self-acceptance and meaning in life

Self-acceptance is defined as an adaptive attitude toward oneself and all characters, which refers to the acceptance of both positive and negative aspects of oneself (Sun and Lu, 2017). Self-acceptance consists of two factors, namely self-judge and self-adoption (Cong and Gao, 1999). Self-judge represents the recognition and the evaluation of the perceived self and self-adoption stands for the subjective acceptance one holds toward one’s perceived self.

As King and Hicks (2021) implied, self-acceptance may contribute to increasing meaning in life and some related literature are listed below. Longitudinal findings showed that college students who feel unoriented from their true selves, indicating a lower level of self-acceptance, tend to be devoid of academic motivation, perceiving all efforts as meaningless and showing a low level of meaning in life (Kim et al., 2018). Accordingly, experimental results also report that participants tend to report a higher level of meaning in life when they are reminded of the characteristics of their truly accepted self, even those flawed ones, which may indicate that the recognition and perceiving self-acceptance may induce the increment of meaning in life (Schlegel et al., 2011). Moreover, self-acceptance was found to share a robust relation with increased positive feelings and life satisfaction, which is identified as the promoter of meaning in life (Miao and Gan, 2019; Liu F. et al., 2021). Indeed, it can be inferred that self-acceptance could be closely related to meaning in life. Additionally, although few studies have explored the symptom-level relations between self-acceptance and meaning in life, one study has found that the relation between self-acceptance and presence of meaning and the relation between self-acceptance and search for meaning differ from each other (Zhou and Xu, 2019), which warrants further exploration.

### 1.3 Social support and meaning in life

Social support represents the status that an individual is cared for, esteemed, and sustained by others or that one has material and psychological resources at one’s disposal (Taylor, 2011). There are three elements in social support, objective social support (the material resources and emotional support offered by the supportive others), subjective social support (the social support including feelings of being esteemed and cared for that the individual actually perceived), and the use of support (the ability to make use of one’s perceived social support) (Xiao, 1999). Social support can bring a lot of benefits, serving as a critical mental resource that keeps individuals both mentally and physically healthy, prompt more engagement in education and at work, and increase the experience that life is meaningful (Zhang et al., 2022; Ma, 2023; Moynihan et al., 2023). One research found that for college students, support from important others, such as mothers and teachers, is a significant source of meaning in life (Li et al., 2022). Based on the structural equation model, Liu et al. (2022) suggested that a lack of social support during the pandemic may lead to enhanced feelings of loneliness and diminished perception of meaning in life. In line with this, Guo S. Q. et al. (2023) also stated that social support could increase college students’ optimism and then contribute to their feelings of meaning in life, which indicated the consistently promoting effect of social support even after the pandemic.

However, the aforementioned study analyzed social support as a whole and they did not differentiate the differences between three factors of social support, which is neither comprehensive nor accurate. As there exists the paradox that those college students confronted with mental challenges are provided with more resources of social support raises the possibility that the third element of social support (Broton et al., 2022), the use of social support, may contribute more to the maintaining of meaning in life. Meanwhile, recent evidence indicates that rural people who may attain less source of objective support report a higher level of meaning in life (Datta and Ostwal, 2023), which could imply the possibility that the influence of objective social support and subjective social support on meaning in life is not equal and warrants further examination.

### 1.4 The current study

To better depict the relation between social support, self-acceptance, and meaning in life, network analysis would be an appropriate approach. Unlike the traditional latent variable model that treats concerned variables as the sum of junior elements, the network analysis allows researchers to look into the relation between different elements of the concerned variables (Tao et al., 2023), identifying key symptoms (i.e., bridge symptoms) among networks (Kaiser et al., 2021). Previous studies have suggested that intervention targeting bridge symptoms among networks may be more efficient (Jones et al., 2021; Wen et al., 2023). Thus, network analysis provides the possibility for deeper understanding and designing more accurate interventions targeting key symptoms in networks (Borsboom and Cramer, 2013; Robinaugh et al., 2020). Indeed, the current study is aimed at filling the gap in the existing literature. Specifically, the present study used network analysis to shed light on the symptom-level relations between meaning in life, self-acceptance, and social support, identifying key symptoms in the network models to improve the level of meaning in life among college students. The following hypotheses were testified:

Aim 1: To better understand the relation between symptoms of social support and self-acceptance, we constructed a network.

Aim 2: Clarify the relation between different factors of meaning in life, social support, and self-acceptance and explore how social support and self-acceptance contribute to two factors of meaning in life.

Hypothesis 1: Considering all indirect evidence stating that individuals with greater self-acceptance may experience more sense of meaning in life (Miao and Gan, 2019; Liu F. et al., 2021), we hypothesized that all symptoms of self-acceptance are positively linked with the search for meaning and presence of meaning.

Hypothesis 2: Previous literature has shown that social support can lead to increased meaning in life, yet people with less material support may report a higher level of meaning in life (Datta and Ostwal, 2023). Indeed, we hypothesized that subjective social support and the use of social support may be positively connected to two components of meaning in life while the relation between objective social support and two factors of meaning in life could be negative.

## 2 Method

### 2.1 Measures

#### 2.1.1 Self-acceptance questionnaire

The self-acceptance questionnaire (SAQ) was developed by Cong and Gao (1999) to measure the level of participants’ self-acceptance. The SAQ contains 16 items which can be divided into two subscales, namely self-acceptance and self-judge. All the items are rated on a 4-point Likert scale, ranging from 1 (*strongly disagree*) to 4 (*strongly agree*) for the items of self-judge and a reverse scoring for items of self-acceptance. The higher level of summed-up scores represents a higher level of self-acceptance. Based on the current participants, the SAQ showed a great internal consistency with *Cronbach’s α* score reaching 0.833 for the whole scale, 0.847 for the self-acceptance subscale, and 0.844 for the self-judge subscale.

#### 2.1.2 Social support rating scale

The social support rating scale (SSRS), developed by Xiao (1999), is aimed at measuring the level of participants’ social support. The SSRS consists of 10 items and includes three subscales, namely objective support (3 items), subjective support (4 items), and the use of support (4 items). Objective support refers to the social supports that actually exist, containing direct material support, social networks, and stable social bonding. Subjective support represents the social support one subjectively perceives, including the feelings of being respected, supported, and understood. The use of support is one’s ability to make the best of the resources of social support, for example, an individual who scored low in this subscale may have adequate resources of social support yet fail to use them. The SSRS was found to be credible and valid among the Chinese population (Yu et al., 2020). In the current study, the *Cronbach’s α* score of SSRS is 0.752.

#### 2.1.3 Meaning in life questionnaire

The Meaning in life questionnaire (MLQ) is a 10-item questionnaire, which was developed to measure participants’ level of meaning in life (Steger et al., 2006) and translated into Chinese by Chen et al. (2015). The MLQ concludes two subscales, namely the presence of meaning (POM) and search for meaning (SFM) and each subscale consists of five items. All items in the MLQ are rated on a 7-point Likert scale where 1 indicates “*absolutely untrue*” and 7 indicates “*absolutely true*.” Each subscale ranges from 5 to 35, with a higher score indicating a higher level of POM or SFM. The MLQ was proved to have good reliability and validity among Chinese college students (Yang et al., 2023). The MLQ shows a good internal consistency reliability, with *Cronbach’s α* score for the whole scale is 0.868, for the POM subscale, 0.851, and for the SFM subscale, 0.889.

### 2.2 Participants and procedure

In the current study, we aimed to explore the contributing factors of meaning in life among college students. Indeed, we used convenience sampling to recruit college students from Southwest University of Science and Technology. The sampling was conducted from July to September in 2023 through the online survey platform, “www.wenjuanxing.com.” The questionnaire was sent to college students by the college counselors and all college students can freely choose to participate in the current study or not. All measurement was shown only after the participants obtained the informed consent. A total of 1,348 participants (Females = 603, *M_age_* = 19.0, *SD_age_* = 3.37, range = 17–27) signed the informed consent and completed the questionnaire. To filter out the careless response, the individual response variability (IRV) of each sample was calculated (Curran, 2016). Samples with an IRV 1.5 quartiles higher than the upper quartile or 1.5 quartiles lower than the lower quartile were identified as careless responders and removed from the dataset. After filtering, there are 1,263 samples (Females = 575, *M_age_* = 19.0, *SD_age_* = 2.59, range = 17–27) left for further analysis. The research was examined and approved by the ethics committee of Beijing Normal University (Number: 202305290090).

### 2.3 Data analysis

#### 2.3.1 Descriptive analysis

In the current study, we used R (version 4.3.2) for the data analysis (Team R.C, 2023). To start with, descriptive analysis to describe the basic information of participants in the current study was conducted. The function *descrTable* was utilized for the description of the data and the generation of the table. After that, the Cronbach’s α scores of each questionnaire and their subscales were calculated to verify the reliability of the scales used in the current study.

#### 2.3.2 Network estimation

To estimate the relation and interaction between factors of social support and self-acceptance, the gaussian graphical model (GGM) was conducted. The GGM is a probabilistic model that represents dependencies between variables using a graph, which could present the relation between multiple variables (Epskamp et al., 2018b). To further regularize the network structure and provide more convenience for understanding, the extended Bayesian information criterion (EBIC) and graphical least absolute shrinkage and selection operator (LASSO) were utilized (Epskamp and Fried, 2018; Epskamp et al., 2018a). Furthermore, we used the package *qgraph* for the visualization of the network (Epskamp et al., 2012). In the network models, variables are characterized as nodes, and the relations between variables are presented as edges. More specifically speaking, in the network the nodes represent the factors of social support and self-acceptance. The green lines linking different nodes stand for that the relation between the correlated nodes is positive and the red lines indicate the opposite. The thicker the lines are the closer the relation is, respectively.

In addition, based on the theoretical hypothesis that social support and self-acceptance may contribute to the generation of meaning in life, we construct two flow network models to clarify how exactly social support and self-acceptance are related to two factors of meaning in life, presence of meaning (POM) and search of meaning (SFM). The flow network is a directed graph that can depict the relation between a certain variable and other multiple variables. In the flow network, each edge represents a pathway through which quantities can move from a source node (e.g., factors of social support) to a sink node (e.g., POM and SFM). Specifically speaking, in the current study, the GGM was used for the estimation and the EBIC as well as the LASSO were utilized for simplification and regularization (Epskamp and Fried, 2018; Epskamp et al., 2018a,b). Differently, package *qgraph* and the function *flow* were employed to present the direct or indirect relation between SFM or POM and factors of social support, and self-acceptance.

#### 2.3.3 The estimation of the centrality index

To clarify the importance and influence of nodes, we calculated the centrality index of the nodes using the function *bridge* and chose Bridge Expected Influence (*BEI*) as the parameter that stands for the relation that one node shared with others. This index is the sum of all positive and negative edge weights that one node connected with other nodes belonging to a different community (e.g., objective support with self-evaluation), which serves as a reliable index to assess the significance of nodes in the network that contains multiple communities (Jones et al., 2021).

#### 2.3.4 Verification of the stability and accuracy of the network

To examine the accuracy and stability of the network models, the R package *bootnet* was employed. To verify the accuracy, we evaluated the bootstrapped confidence intervals (95% *CI*s) by using the nonparametric bootstrap. In this part, the narrower the *CIs* are, the more accurate and reliable the network models are. Moreover, to test whether there exists a significant difference between edge weights and *BEI*, the bootstrapped difference test was employed. In addition, to testify to the stability of the network, the correlation stability coefficients (*CS-C*) of the *BEI* were calculated, which refers to the maximum proportion of the sample that can be removed while the correlation coefficient of the *BEI* among the original sample and the after-dropped sample still reach at least 0.7 at the probability of 95% (Epskamp et al., 2018a). As a previous study suggested, the *CS-C* should be at least 0.25 and is preferable than 0.5 (Cheung et al., 2021).

## 3 Result

The descriptive information and basic demographic information of the participants in the current study are shown in Table 1.

**Table 1:** The descriptive information (*N* = 1,263).

	*Mean (SD)*	*Skew*	*Kurtosis*	*N*
Gender				1,263
Boy	668 (54.5%)			
Girl	575 (45.5%)			
Age	19.0 (2.59)	23.587	722.09	1,263
Social support	36.4 (7.22)	0.116	0.248	1,263
Objective support	8.17 (2.73)	0.412	0.834	1,263
Subjective support	20.78 (4.71)	0.217	−0.310	1,263
Use of support	7.43 (2.00)	0.213	−0.025	1,263
Self-acceptance	38.3 (6.64)	−0.336	0.928	1,263
Self-acceptance	19.1 (4.43)	−0.011	0.330	1,263
Self-judge	19.3 (4.17)	−0.023	0.338	1,263
Meaning in life	49.9 (9.87)	−0.050	0.468	1,263
Presence of meaning	23.2 (6.07)	−0.041	0.167	1,263
Search for meaning	26.7 (5.75)	−0.461	0.336	1,263

### 3.1 Network structure

**Figure 1:**
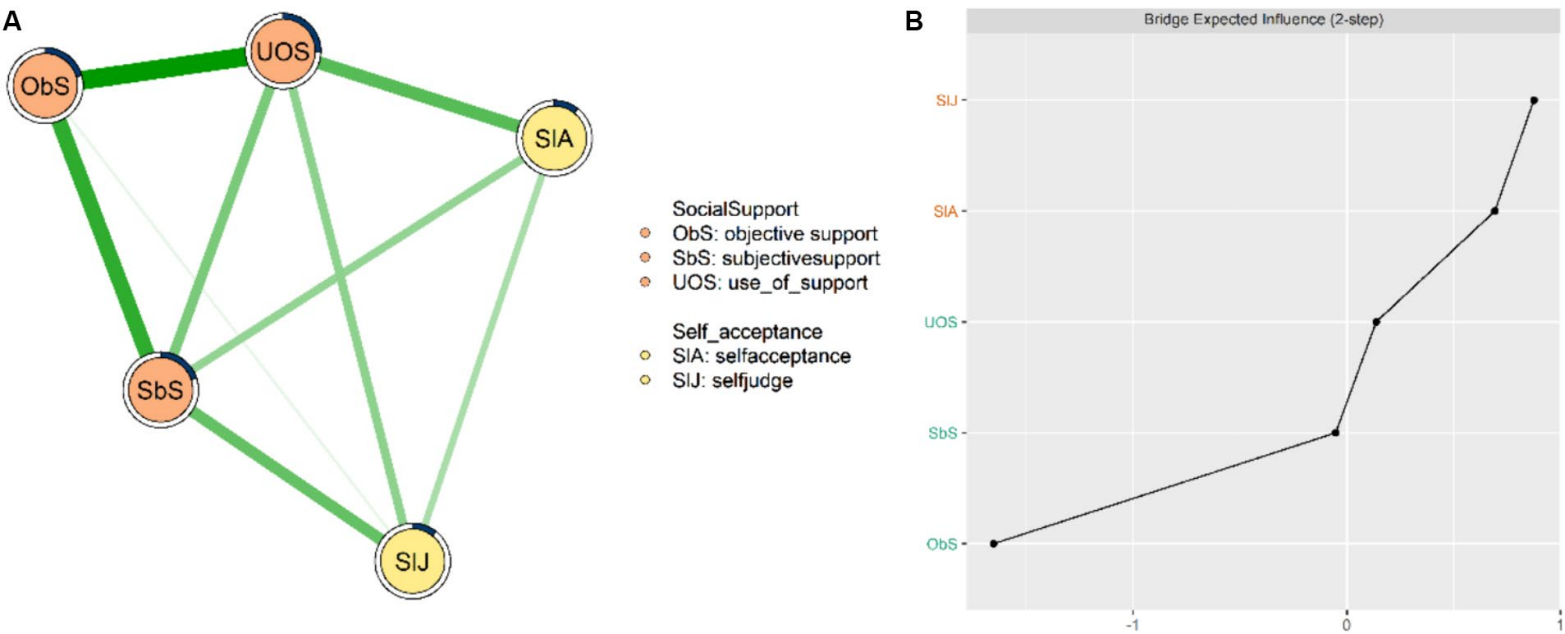
Network structures and standardized *BEI*. **(A)** The network model of social support and self-acceptance with age and gender controlled as covariates. **(B)** The standardized *BEI* of each node.

The network model of social support and self-acceptance is depicted in Figure 1A. There are five nodes in this network structure, with 9 edges identified as non-zero edges (90%). Figure 1B shows the rank of the *BEI* of the nodes in the network model and “Self-acceptance” served as the node with the highest *BEI* value (*BEI* = 0.461) among all nodes. Meanwhile, the results also indicate that the top three strongest edges are “Objective support”- “Use of Support” (*edge weight* = 0.268, 95% *CI* covering the range of 0.216–0.320, *p* < 0.001), “Subjective support”- “Objective support” (*edge weight* = 0.218, 95% *CI* covering the range of 0.161–0.276, *p* < 0.001), and “Self-acceptance”- “Use of Support” (*edge weight* = 0.177, 95% *CI* covering the range of 0.115–0.239, *p* < 0.001) (see Supplementary Table S1).

### 3.2 Flow network

**Figure 2:**
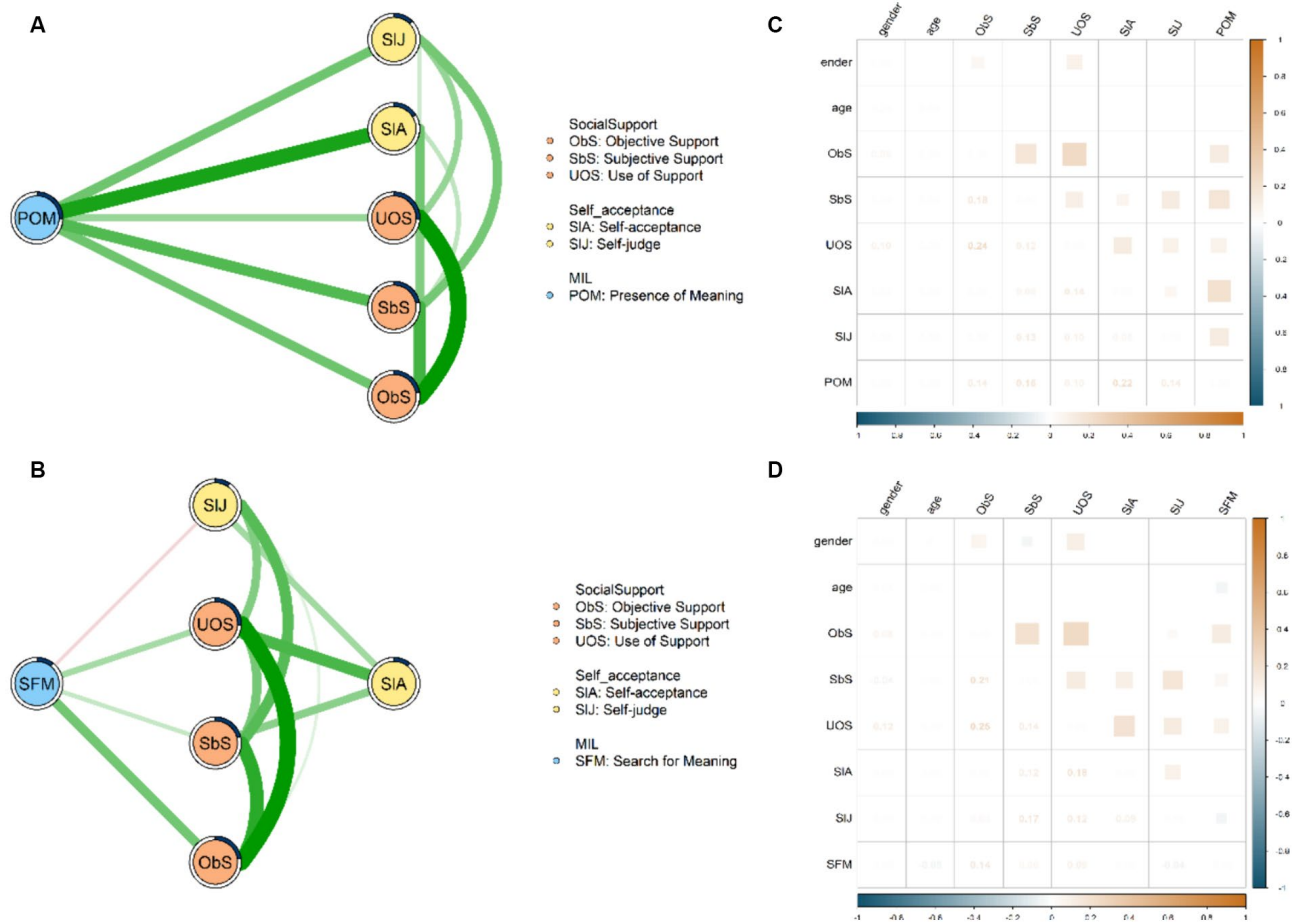
Flow network structures and relation between different nodes with age and gender controlled as covariates. **(A)** The flow network model of POM, social support, and self-acceptance. **(B)** The flow network model of SFM, social support, and self-acceptance. **(C)** The relation between different nodes in the network of POM, social support, and self-acceptance as well as the covariates. **(D)** The relation between different nodes in the network of SFM, social support, and self-acceptance as well as the covariates.

The flow network model of POM, self-acceptance, and social support is shown in Figure 2A. The flow network model contains six nodes, with 13 edges out of 15 edges (86.7%) determined as non-zero edges. Among all the non-zero edges connecting to “POM,” “POM”- “Self-acceptance” (*edge weight* = 0.216, 95% *CI* covering the range of 0.156–0.277, *p* < 0.001), “POM”- “Subjective support” (*edge weight* = 0.163, 95% *CI* covering the range of 0.107–0.218, *p* < 0.001), and “POM”- “Self-judge” (*edge weight* = 0.136, 95% *CI* covering the range of 0.078–0.195, *p* < 0.001) are the top three strongest (see Figure 2C, for more detailed information, see Supplementary Table S2). Meanwhile, Figure 2B demonstrates the flow network model of POM, self-acceptance, and social support. This flow network model includes six nodes and 13 non-zero edges out of a total of 15 edges (86.7%). Considering the non-zero edges linking “SFM” with other nodes, “SFM”- “Objective support” (*edge weight* = 0.140, 95% *CI* covering the range of 0.082–0.198, *p* < 0.001), “SFM”- “Use of support” (*edge weight* = 0.089, 95% *CI* covering the range of 0.032–0.146, *p* < 0.01), and “SFM” – “Subjective support” (*edge weight* = 0.058, 95% *CI* covering the range of −0.002-0.118, *p* = 0.099) are identified as the top three strongest edges (see Figure 2D, for more detailed information, see Supplementary Table S3).

### 3.3 Network stability and accuracy

The results of the bootstrapped analysis are demonstrated in Supplementary Figure S1. As shown in Supplementary Figure S1A, the three top strongest edges in the network model of social support and self-acceptance are significantly different from the other edges. Meanwhile, Supplementary Figure S1B also indicates a significant difference between the *BEI* values of the nodes in the network model. Similarly, the edge weights and *BEI* values in the flow network models of two different factors of meaning in life and social support, self-acceptance are also significantly different from the other edges and nodes (see Supplementary Figure S2 for the flow network model of POM, social support, and self-acceptance, see Supplementary Figure S3 for the flow network model of SFM, social support, and self-acceptance).

The results of case dropping indicate that all three network models are stable. The *CS-Cs* of *BEI* for the network model of social support and self-acceptance reached 0.75, while for the flow network model of POM, social support, and self-acceptance, it also reached 0.75. For the flow network model of SFM, social support, and self-acceptance, it reached 0.672. The results of case dropping are shown in Supplementary Figure S4A for the network model of social support and self-acceptance, Supplementary Figure S4B for the flow network model of POM, social support, and self-acceptance, and Supplementary Figure S4C for the flow network model of SFM, social support, and self-acceptance.

## 4 Discussion

The present study aimed at exploring the symptom-level relations between self-acceptance and social support. Furthermore, we also contributed efforts to find out the relations between symptoms of self-acceptance and social support and the two subdimensions of MLQ: POM and SFM. Several important findings need to be elucidated.

In the cross-sectional symptom network of self-acceptance and social support, we found that the symptom “SIA” (Self-acceptance) served as the bridge symptom. In other words, “SIA” (Self-acceptance), the emotional and attitudinal acceptance of the actual self (Cong and Gao, 1999), is the symptom that connects self-acceptance and social support. There are two possible explanations for this result. For one thing, our results may suggest that social support that can elevate the emotional and attitudinal acceptance of their actual self will be more useful in improving self-acceptance. For another, people who accept themselves emotionally and attitudinally may obtain more social support. Previous literature identified that people with high self-acceptance were more likely to employ positive coping strategies (Komarudin et al., 2022). Seeking partial and emotional support proactively is a positive coping strategy for facing difficulties (Freire et al., 2016) and individuals with high levels of “SIA” (Self-acceptance) may be proactive in seeking support.

Even though the causal relations between social support and self-acceptance are unexplored for the limitations of cross-sectional design, the findings of the present study highlighted the significance of the symptom “SIA” (Self-acceptance). As previous research documented, self-acceptance and social support are internal and external protective factors of mental health, respectively (Huang et al., 2020). Interventions targeting in improving “SIA” (Self-acceptance) may be effective in helping individuals cultivate social support system and self-acceptance and improve their mental health.

The current research constructed the flow network models of POM and SFM, separately. In the flow network models, we found that all symptoms of self-acceptance are positively related to POM and SFM, supporting Hypothesis 1. In terms of the relations between symptoms of social support, POM, and SFM among college students, analysis of the current study did not support Hypothesis 2. Even though symptoms of social support have positive associations with both POM and SFM, the edge values of symptom-level associations between social support and meaning in life differed across two flow network models. Specifically, in the flow network of POM, “SIA” (Self-acceptance) has the strongest direct and positive association with POM. The connection between “SbS” (Subjective Support) and POM is the second strongest of all connections. Differently, “ObS” (Objective Support) shares the strongest positive association with SFM and “UOS” (Use of Support) and “SbS” (Subjective Support) are the second and third, respectively. These findings imply three important points.

First, the discrepancies between the flow network of POM and SFM may imply that despite both being measures of meaning in life, POM and SFM have intrinsic differences. This is supported by a wealth of literature. For example, one meta-analysis containing 147 studies showed that the relations between POM and subjective well-being and SFM and subjective well-being were different. POM and subjective well-being had a stronger positive association while SFM and subjective well-being had a smaller and negative association (Li et al., 2021). Yek et al. (2017) study found that higher POM was associated with lower health anxiety but the opposite relation was observed for SFM and health anxiety. These studies, along with our findings, imply that it may be necessary to further differentiate between the two dimensions when examining the meaning in life, considering that the two dimensions are different and sometimes have even contradictory effects.

Second, the results of flow network models highlight the significance of improving “SIA” (Self-acceptance) when we try to enhance the meaning of life among college students. Even though, to our best knowledge, no previous study examines the relations between different dimensions of meaning in life, symptoms of self-acceptance, and symptoms of social support, plenty of studies have tried to explore the sources of meaning in life. These studies may explain the significance of “SIA” (Self-acceptance) found in this study. Factors such as community activities, personal development and relationships with others have positive relations with not only POM, but also SFM (Grouden and Jose, 2015). Dewitte et al. (2021) analyzed several variables that are directly linked with POM and found that among variables such as personal growth, spirituality, and interpersonal relationships, the strongest associations existed between personal growth and POM. What’s more, the participants of the current study were all college students, most of whom were facing important life decisions such as career planning and were in the transition stage from school to society (Renn et al., 2014). This may also explain the finding that the symptom most strongly associated with POM in this study was “SIA” (Self-acceptance). Grouden and Jose (2014) also identified that younger individuals were more likely to find personal growth meaningful, consistent with the current finding.

Third, the results of flow network models highlight the role of social support among college students. The analysis of the current study found that three dimensions of social support are positively linked with both POM and SFM. Additionally, although previous studies have found that objective support may be negatively associated with meaning in life (Datta and Ostwal, 2023), this phenomenon was not found in this study. This may be due to the fact that the college period is an important transition period (Medalie, 1981), during which they have limited individual abilities and are also more likely to encounter financial stress, anxiety, and other problems (Beiter et al., 2015). Therefore, social support is important at this stage, both for college students’ POM and SFM. In other words, adequate social support may provide college students with more courage to explore the meaning of life.

### 4.1 Implications

The findings of this research have several practical implications. First, this study highlights the significance of symptoms “SIA” (Self-acceptance). This research suggests that improving the level of “SIA” (Self-acceptance) will do good to both social support (especially increasing use of support) and meaning in life. Interventions such as cognitive behavioral therapy and paint therapy group counseling can be implemented to improve self-acceptance among college students (Pasaribu and Zarfiel, 2018; Zheng et al., 2021). Second, the findings of this research imply that social support plays an important role in enhancing the meaning of life for college students. Colleges can employ support group intervention, which has positive impacts on social support among college students (Lamothe et al., 1995; Mattanah et al., 2012).

### 4.2 Limitations

To our best knowledge, the present research is the first research to explore the symptom-level relations between self-acceptance, social support, and meaning in life, providing fresh insights into understanding the complex associations between the aforementioned variables. However, several deficits of the current study still need to be noted. First, the current study uses a cross-sectional design, not allowing causal conclusions to be drawn. Thus, future studies can employ longitudinal studies to explore the complex causal relations between the symptoms of these variables. Second, this study utilized self-report questionnaires to measure self-acceptance, social support, and meaning in life, which is inevitably affected by the daily emotions of participants and the social desirability bias. To mitigate this limitation, future research can employ experiments or add objective indicators to explore the relations between social support, self-acceptance, and meaning in life. Third, age, cognitive styles, and personality would affect the meaning in life (Allan et al., 2015; Pang et al., 2019) and the associations between social support and self-acceptance. Even though we controlled the effect of age and gender in the current study, it should be cautious when generalizing the results of this study to other samples. More studies are needed to validate the results of the current study among other samples.

## 5 Conclusion

The current study explored the relationship between self-acceptance, social support, and meaning in life using symptom network analysis with college students as subjects. The analysis found that “SIA” (Self-acceptance) was the key bridge symptom in the symptom network of self-acceptance and social support and it can be an important targeted symptom when improving both social support and self-acceptance. According to the results of flow networks, all symptoms of social support and self-acceptance are positively related to meaning in life. The analysis of flow network models of POM and SFM showed that “SIA” (Self-acceptance) and “ObS” (Objective Support) are the nodes with the strongest positive relationship with POM (or SFM) in the two networks, respectively.

## Data availability statement

The raw data supporting the conclusions of this article will be made available by the authors, without undue reservation.

## Ethics statement

The studies involving humans were approved by The research was examined and approved by the ethics committee of Beijing Normal University (Reference number: 202305290090). The studies were conducted in accordance with the local legislation and institutional requirements. The participants provided their written informed consent to participate in this study.

## Author contributions

CW: Formal analysis, Investigation, Writing – original draft, Methodology, Software. XL: Investigation, Writing – review & editing. JL: Investigation, Writing – review & editing, Data curation. YT: Conceptualization, Methodology, Project administration, Writing – review & editing. YL: Conceptualization, Funding acquisition, Resources, Validation, Writing – review & editing.
