# Supplementary material for: Strengthening the meaning in life among college students: the role of self-acceptance and social support - evidence from a network analysis
Source: Front Psychol. 2024 Jul 15;15:1433609. doi: 10.3389/fpsyg.2024.1433609 (PMC11284095; doi:10.3389/fpsyg.2024.1433609)
Supplement: Supplementary file 1 [file Data_Sheet_1.docx]

**Table S1** The weights, CIlower, CIupper and significance level of edges in the network of social support and self-acceptance

| ***Edge*** | ***weight*** | ***CIlower*** | ***CIupper*** | ***p*** |
| --- | --- | --- | --- | --- |
| age--objective support | 0 | -0.04212 | 0.042119 | 0.643 |
| age--self-acceptance | 0 | -0.0169 | 0.016899 | 0.934 |
| age—self-judge | 0 | -0.02003 | 0.020032 | 0.918 |
| age--subjective support | 0 | -0.01981 | 0.019806 | 0.922 |
| age--use of support | 0 | -0.02195 | 0.021947 | 0.88 |
| gender--age | 0 | -0.02747 | 0.027468 | 0.782 |
| gender--objective support | 0.068476 | 0.009086 | 0.127867 | 0.038 |
| gender--self-acceptance | 0 | -0.02213 | 0.022131 | 0.881 |
| gender—self-judge | 0 | -0.01594 | 0.015936 | 0.929 |
| gender--subjective support | -0.02451 | -0.08012 | 0.031109 | 0.547 |
| gender--use of support | 0.11157 | 0.054101 | 0.16904 | 0 |
| objective support--self-acceptance | 0 | -0.01536 | 0.015362 | 0.89 |
| objective support—self-judge | 0.024736 | -0.02445 | 0.073923 | 0.259 |
| objective support--subjective support | 0.218262 | 0.16056 | 0.275963 | 0 |
| objective support--use of support | 0.268129 | 0.215857 | 0.320402 | 0 |
| self-acceptance—self-judge | 0.088995 | 0.007894 | 0.170096 | 0.032 |
| subjective support--self-acceptance | 0.112705 | 0.052445 | 0.172964 | 0.002 |
| subjective support—self-judge | 0.162116 | 0.100688 | 0.223545 | 0 |
| subjective support--use of support | 0.139306 | 0.079568 | 0.199044 | 0 |
| use of support--self-acceptance | 0.177057 | 0.115471 | 0.238643 | 0 |
| use of support—self-judge | 0.118824 | 0.061275 | 0.176374 | 0 |

**Table S2** The weights, CIlower, CIupper and significance level of edges in the flow network of POM, social support, and self-acceptance

| ***Edge*** | ***Weights*** | ***CIlower*** | ***CIupper*** | ***p*** |
| --- | --- | --- | --- | --- |
| age— POM | 0 | -0.00761 | 0.007605 | 0.974 |
| age--objective support | 0 | -0.04088 | 0.04088 | 0.65 |
| age--self-acceptance | 0 | -0.01537 | 0.015372 | 0.929 |
| age—self-judge | 0 | -0.02076 | 0.02076 | 0.881 |
| age--subjective support | 0 | -0.01713 | 0.017131 | 0.918 |
| age—use of support | 0 | -0.02239 | 0.022394 | 0.862 |
| gender— POM | 0 | -0.05002 | 0.050017 | 0.534 |
| gender--age | 0 | -0.02697 | 0.026967 | 0.763 |
| gender--objective support | 0.052176 | -0.00747 | 0.111818 | 0.019 |
| gender--self-acceptance | 0 | -0.01442 | 0.01442 | 0.924 |
| gender—self-judge | 0 | -0.01619 | 0.016194 | 0.922 |
| gender--subjective support | 0 | -0.04947 | 0.049472 | 0.544 |
| gender—use of support | 0.096097 | 0.038756 | 0.153437 | 0.001 |
| objective support— POM | 0.136445 | 0.07831 | 0.19458 | 0 |
| objective support--self-acceptance | 0 | -0.03373 | 0.033726 | 0.859 |
| objective support--self judge | 0 | -0.03546 | 0.03546 | 0.534 |
| objective support--subjective support | 0.178369 | 0.121284 | 0.235455 | 0 |
| objective support—use of support | 0.240094 | 0.182791 | 0.297398 | 0 |
| self-acceptance— POM | 0.216319 | 0.155705 | 0.276933 | 0 |
| self-acceptance—self-judge | 0.047819 | -0.02696 | 0.122597 | 0.106 |
| Self-judge— POM | 0.136019 | 0.076155 | 0.195883 | 0 |
| subjective support— POM | 0.162512 | 0.10663 | 0.218394 | 0 |
| subjective support--self-acceptance | 0.060155 | 0.002014 | 0.118297 | 0.024 |
| subjective support—self-judge | 0.12917 | 0.067279 | 0.191061 | 0 |
| subjective support—use of support | 0.117529 | 0.057872 | 0.177186 | 0 |
| Use of support— POM | 0.097353 | 0.041662 | 0.153045 | 0 |
| Use of support--self-acceptance | 0.135105 | 0.075617 | 0.194593 | 0 |
| Use of support—self-judge | 0.098907 | 0.04156 | 0.156254 | 0 |

**Table S3** The weights, CIlower, CIupper and significance level of edges in the flow network of SFM, social support, and self-acceptance

| ***Edge*** | ***Weight*** | ***CIlower*** | ***CIupper*** | ***p*** |
| --- | --- | --- | --- | --- |
| age--SFM | -0.05108 | -0.09706 | -0.00511 | 0.183 |
| age—objective support | 0 | -0.03132 | 0.031317 | 0.714 |
| age--self-acceptance | 0 | -0.01664 | 0.016641 | 0.932 |
| age—self-judge | 0 | -0.01666 | 0.016664 | 0.92 |
| age--subjective support | 0 | -0.01459 | 0.01459 | 0.943 |
| age--use of support | 0 | -0.01975 | 0.019751 | 0.863 |
| gender--SFM | 0 | -0.01987 | 0.019868 | 0.913 |
| gender--age | 0.008404 | -0.01518 | 0.031983 | 0.794 |
| gender--objective support | 0.076916 | 0.020883 | 0.13295 | 0.03 |
| gender--self-acceptance | 0 | -0.02037 | 0.020371 | 0.906 |
| gender—self-judge | 0 | -0.01393 | 0.013929 | 0.942 |
| gender--subjective support | -0.04036 | -0.08999 | 0.009259 | 0.561 |
| gender--use of support | 0.119784 | 0.063642 | 0.175927 | 0 |
| objective support--SFM | 0.139999 | 0.081612 | 0.198386 | 0 |
| objective support--self-acceptance | 0 | -0.01589 | 0.015889 | 0.874 |
| objective support--self judge | 0.033031 | -0.0162 | 0.082258 | 0.249 |
| objective support--subjective support | 0.213067 | 0.156107 | 0.270026 | 0 |
| objective support--use of support | 0.254676 | 0.202158 | 0.307193 | 0 |
| self-acceptance--SFM | 0 | -0.02103 | 0.021029 | 0.88 |
| self-acceptance—self-judge | 0.093272 | 0.013619 | 0.172926 | 0.021 |
| Self-judge--SFM | -0.04228 | -0.10274 | 0.018181 | 0.544 |
| subjective support--SFM | 0.058079 | -0.00183 | 0.117985 | 0.099 |
| subjective support—self-acceptance | 0.116283 | 0.054744 | 0.177822 | 0 |
| subjective support--self judge | 0.168002 | 0.107218 | 0.228787 | 0 |
| subjective support--use of support | 0.136838 | 0.079448 | 0.194228 | 0 |
| use of support--SFM | 0.088924 | 0.031549 | 0.146299 | 0.005 |
| use of support--self-acceptance | 0.180649 | 0.122477 | 0.238822 | 0 |
| use of support—self-judge | 0.124719 | 0.064947 | 0.18449 | 0 |


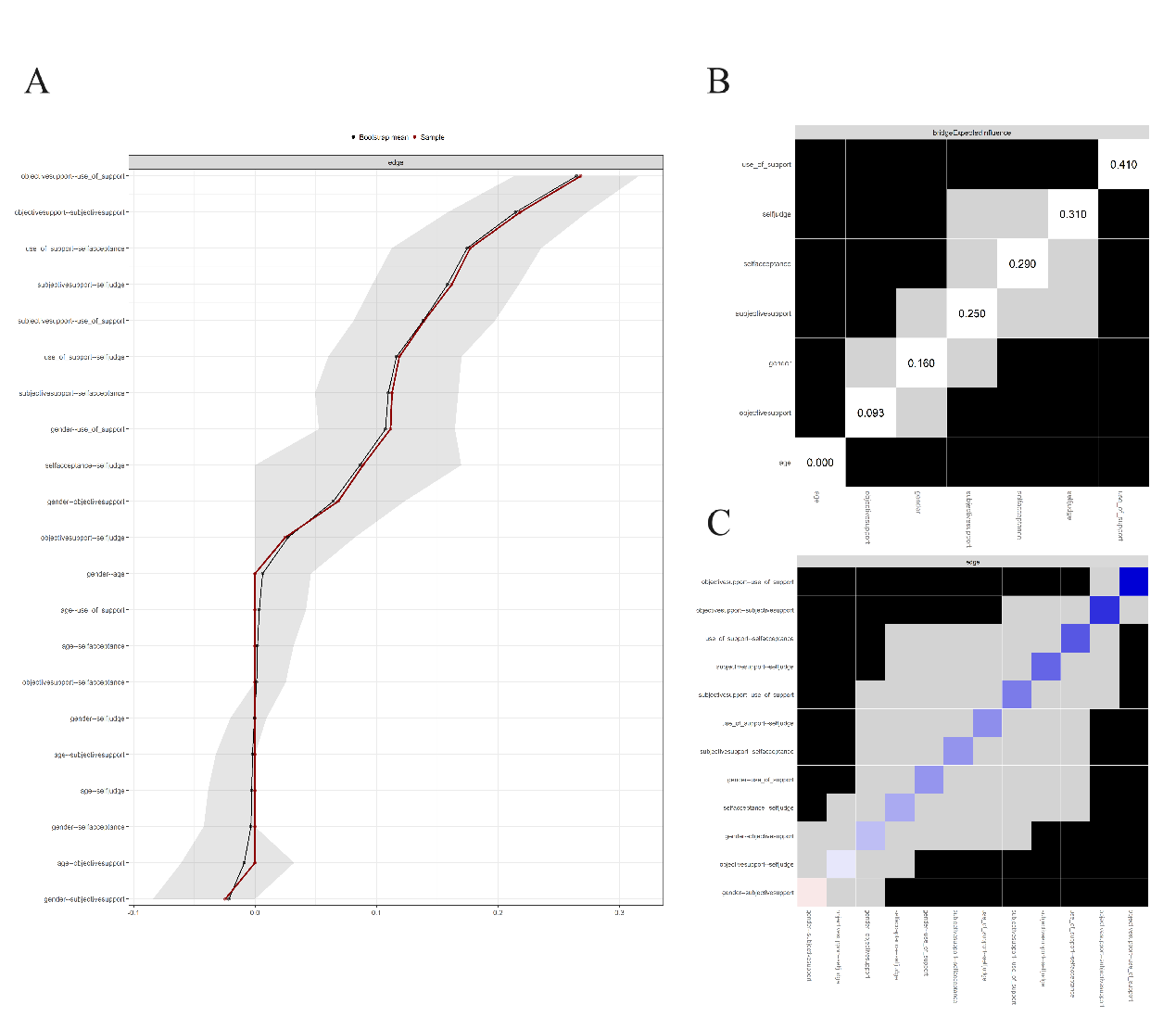


**Figure S1.** Nonparametric bootstrapped difference test for edges and bridge EI values as well as the nonparametric bootstrapped confidence intervals of estimated edges for the network model of social support and self-acceptance. A, Confidence intervals of estimated edges. The red line represents the estimated edge, while the dark area indicates the 95% bootstrap confidence interval. B, *BEI* values. B, *Edge weights*. Grey boxes indicate no significant difference, whereas black box indicate a statistically significant difference (*p* < 0.05). Diagonal color and saturation represent the magnitude and direction of each estimated edge.


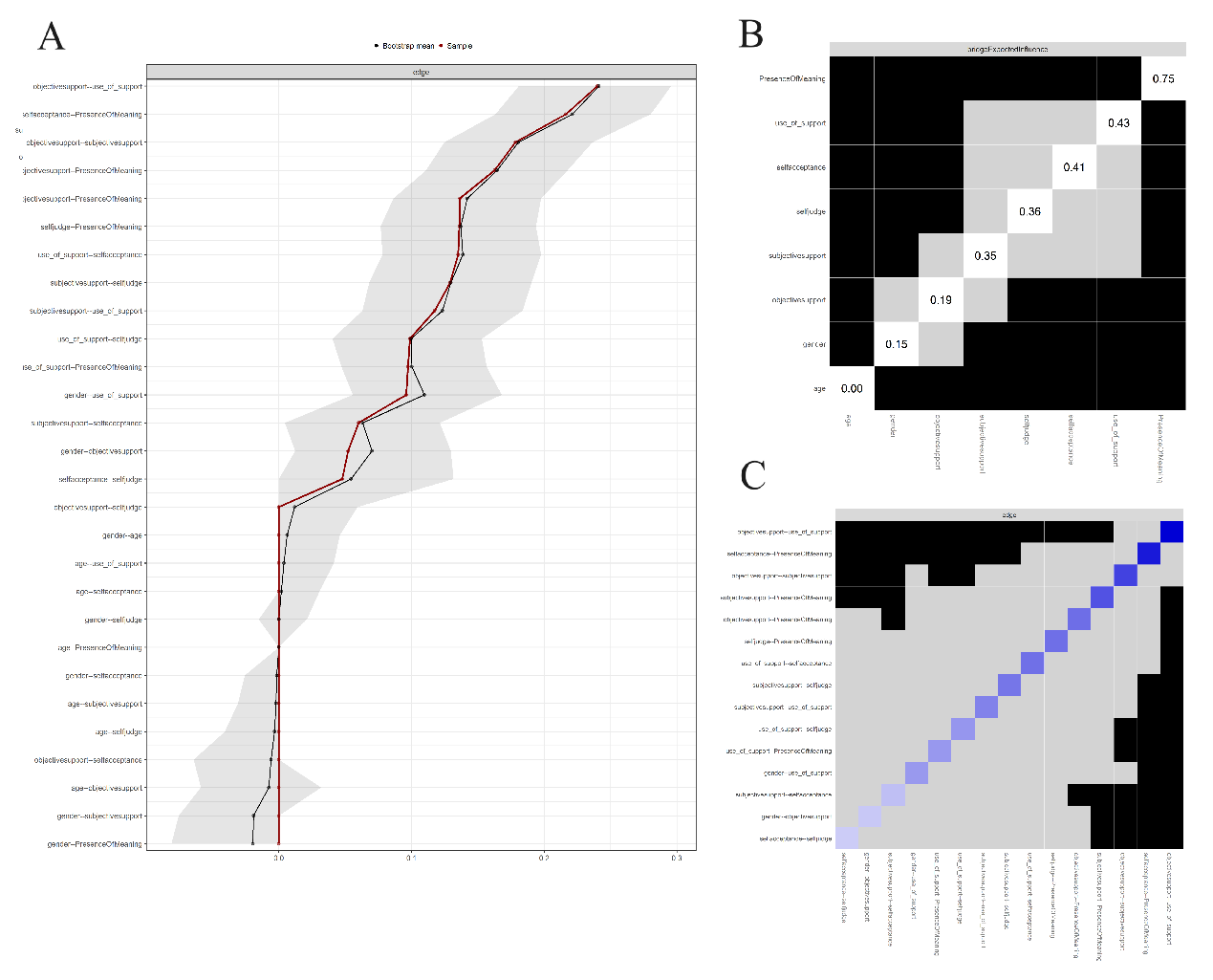


**Figure S2.** Nonparametric bootstrapped difference test for edges and bridge EI values as well as the nonparametric bootstrapped confidence intervals of estimated edges for the flow network model of POM, social support, and self-acceptance. A, Confidence intervals of estimated edges. B, *BEI* values. C, *Edge weights*.


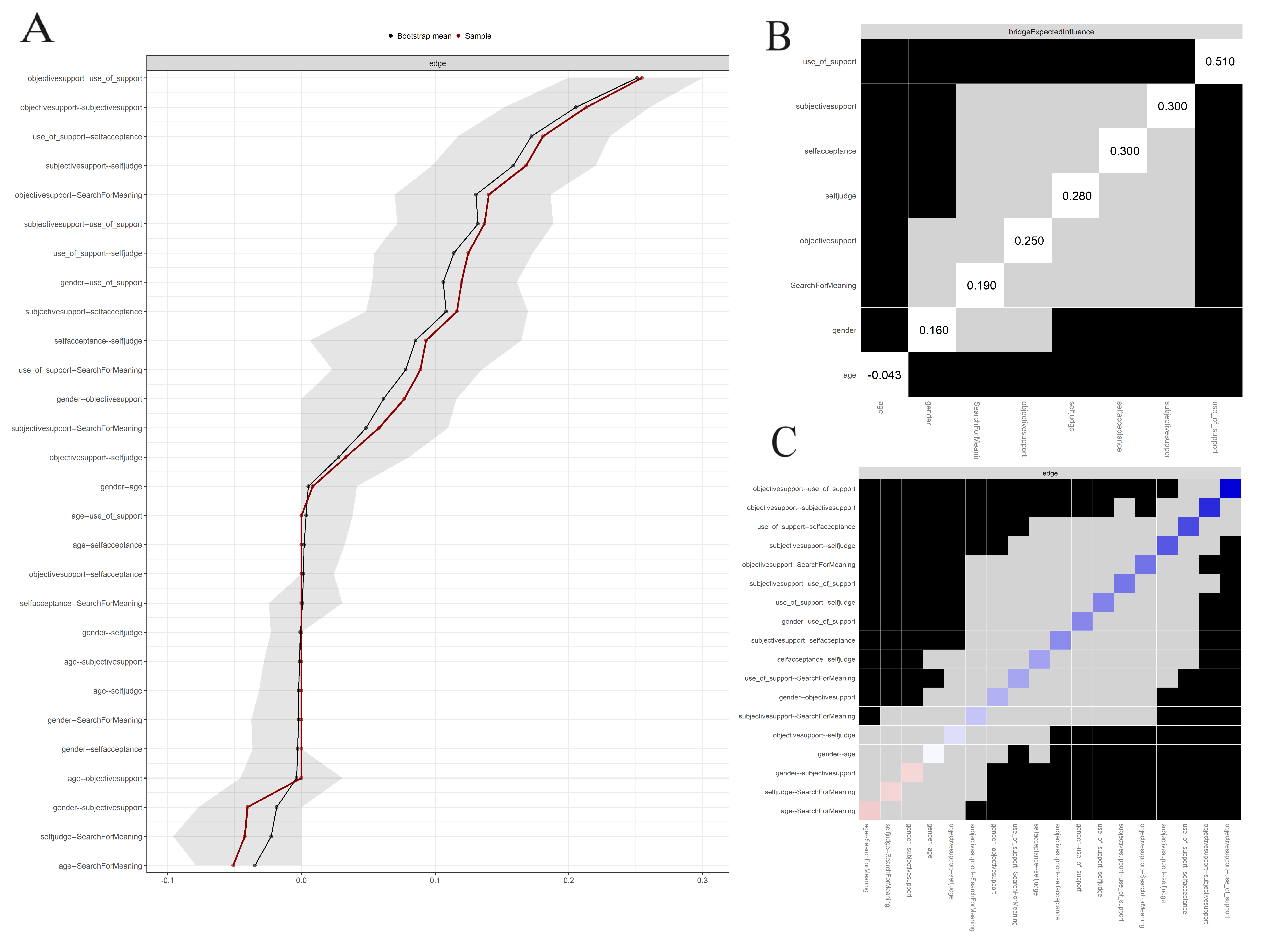


**Figure S3.** Nonparametric bootstrapped difference test for edges and bridge EI values as well as the nonparametric bootstrapped confidence intervals of estimated edges for the flow network model of SOM, social support, and self-acceptance. A, Confidence intervals of estimated edges. B, *BEI* values. C, *Edge weights*.


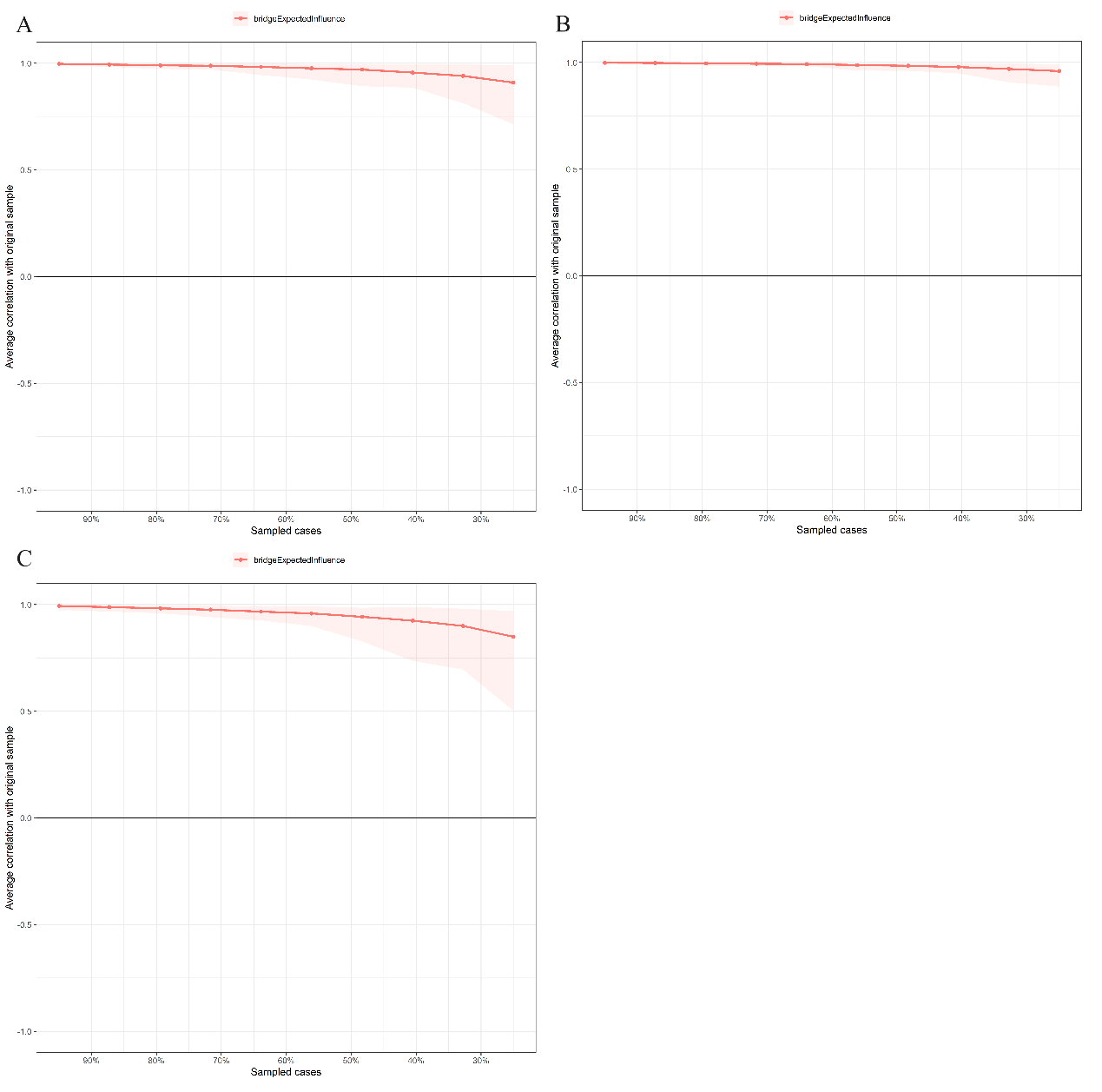


**Figure S4.** Case-dropping analysis of bridge expected influence. The x-axis indicates the percentage of cases of the original sample included at each step. The y-axis indicates the average of correlations between the centrality indices from the original network and the centrality indices from the networks that were re-estimated after excluding increasing percentages of cases. A, network model of social support and self-acceptance. B, flow network model for POM, social support, and self-acceptance. B, flow network model for SOM, social support, and self-acceptance.
